# Oxford Nanopore Sequencing Reveals an Exotic *Broad bean mottle virus* Genome within Australian Grains Post-Entry Quarantine

**DOI:** 10.1128/mra.00211-22

**Published:** 2022-05-31

**Authors:** S. Maina, S. L. Norton, V. L. McQueen, S. King, B. Rodoni

**Affiliations:** a Australian Grains Genebank, Agriculture Victoria Research, Horsham, Victoria, Australia; b Elizabeth Macarthur Agricultural Institute, NSW Department of Primary Industries, Menangle, New South Wales, Australia; c Microbial Pests & Diseases, Agriculture Victoria Research, AgriBio, Bundoora, Victoria, Australia; d School of Applied Systems Biology, La Trobe University, Bundoora, Victoria, Australia; KU Leuven

## Abstract

A *Broad bean mottle virus* (BBMV) isolate (S52) obtained from an infected Vicia faba leaf sample from Syria was sequenced using Oxford Nanopore long-read sequencing at the Australian border. The genome had 95.6%, 98.2%, and 93.4% nucleotide sequence identity to BBMV strains RNA1 (Bawden), RNA2 (Mo), and RNA3 (Bawden).

## ANNOUNCEMENT

*Broad bean mottle virus* (BBMV) (*Bromovirus*, *Bromoviridae*) is a tripartite linear single-stranded RNA (ssRNA) (+) seed-borne virus that infects several temperate pulses. It is found in Africa, Asia, Europe, and the Middle East, where it causes severe damage in pulses (1–3) and constitutes a threat to crop improvement programs (4). BBMV is a quarantinable pathogen in Australia; consequently, it is mandatory for the imported seeds to be screened within post-entry quarantine (PEQ) (5).

Isolate S52 was obtained from Vicia faba (faba bean) material imported from Syria in 2003 and mechanically inoculated into faba bean plants in a PEQ controlled glasshouse. The inoculated plants revealed mottling and chlorotic blotch symptoms; the leaves were sampled and tested positive for BBMV by tissue blot immunoassay (6). Total RNA was extracted from the positive leaf material using a ZR plant RNA miniprep kit (Zymo Research), and a quality control check was conducted as previously described (7). The RNA was converted to cDNA using random primers (8), followed by library preparation using a ligation sequencing kit (SQK-LSK109; ONT); the sequencing was conducted on the MinION platform using a FLO-MIN106D (R9.5) flow cell. Base calling was performed using MinKNOW version 20.10.3 in high accuracy mode. A total of 101,000 reads were generated, ranging in length from 1.2 to 5.3 kb, and imported into CLC Genomics Workbench (CLCGW) version 20 (CLC bio; Qiagen) for quality control, followed by *de novo* assembly using default settings with double polishing. In addition, the reads were mapped onto a reference BBMV genome using Minimap2 version 2.0.0 (9).

The consensus genome segments had 351 to 610 reads mapping onto each entire coding genome segment, with average depths of 22×, 31×, and 48× and GC contents of 43%, 42%, and 44% for RNA1 to RNA3, respectively. The consensus sequence was subjected to a BLASTN search using BLAST+ version 2.7 and MUSCLE (10, 11). The new genome had 95.6%, 98.2%, and 93.4% nucleotide identity (nt) to BBMV strains RNA1 (Bawden), RNA2 (Mo), and RNA3 (Bawden), respectively (12, 13). Primers were designed from the newly generated BBMV RNA3 coat protein (CP) region, targeting 250 to 500 bp, and amplified the expected amplicon band size. Annotation of the BBMV genome (5) revealed that both RNA1 and RNA2 encoded open reading frames 1a and 2a, typical of the genus *Bromovirus*, to which BBMV belongs (13). Further, RNA3 encoded the CP gene, which is required for virus systemic movement and associated with cell-to-cell spread (14). The 93.4% nt between S52 and Bawden suggests that the CP might be a hotspot for BBMV genome variability. Nevertheless, considering the data quality score (15), this deduction should be confirmed further by Illumina reads.

BBMV vectors have not been reported in Australia, and imported germplasm remains the major introduction pathway. Imported seeds serve as a source of genetic diversity for Australian grain-breeding programs. Therefore, it is plausible that the importation of this new germplasm into Australia poses a significant threat in introducing damaging exotic viruses and their variants. As such, persistent surveillance and the integration of robust diagnostic genomics tools at the PEQ border are vital in safeguarding the Australian grains industry.

### Data availability.

The data described here were deposited in the DDBJ under accession numbers LC683787 to LC683789. The raw reads were deposited at the SRA under accession number SRR16883245 and BioProject accession number PRJNA778895.
